# Can we improve the diagnosis of invasion in encapsulated follicular-patterned thyroid tumors? Data from a massive international e-learning initiative

**DOI:** 10.1007/s00428-025-04045-1

**Published:** 2025-02-24

**Authors:** Yara Mansour, Léonor Chaltiel, Ana Cavillon, Abderrahmane Al Bouzidi, Bakarou Kamate, Antonio De Leo, Andriamampihantona Lalaoarifetra Ramiandrasoa, Karima Mrad, Nassim Vibert, Manuel Sobrinho-Simoes, Giovanni Tallini, Philippe Vielh, Geneviève Belleannée

**Affiliations:** 1 Department of Pathology, Haut-Leveque Hospital, University Medical Centre, Bordeaux, France; 2 Biostatistics Unit, Institut Claudius Régaud, Institut Universitaire du Cancer de Toulouse - Oncopole Toulouse, Toulouse, France; 3 Department of Pathology Cheikh, Faculty of Medicine, Khalifa International Hospital, University Mohammed VI of Health Sciences, Casablanca, Morocco; 4 Faculty of Medicine and Odontostomatology, University of Sciences Techniques and Technologies of Bamako, Bamako, Mali; 5 Anatomic Pathology, Department of Medical and Surgical Sciences (DIMEC), University of Bologna Medical Center, Bldg. 20 - Policlinico S.Orsola, Room 020+4A 004, Via Massarenti 9, Bologna, 40138 Italy; 6 Solid Tumor Molecular Pathology Laboratory, IRCCS Azienda Ospedaliero, Universitaria Di Bologna, Bldg. 20 - Policlinico S.Orsola, Room 020+4A 004, Via Massarenti 9, Bologna, 40138 Italy; 7 Anatomic Pathology, Malagasy Lutheran Health Department (SALFA), Antananarivo, Madagascar; 8 Pathology Department, Faculty of Medicine, Salah Azaiez Institute, El Manar University, Tunis, Tunisia; 9 Radiotherapy Department, Bergonié Institute, Bordeaux, France; 10 Department of Pathology, Institute of Molecular Pathology and Immunology (IPATIMUP), University of Porto, Porto, Portugal; 11 Medipath and American Hospital of Paris, Paris, France

**Keywords:** Thyroid tumors, Follicular pattern, Capsular invasion, Vascular invasion, E-learning

## Abstract

With the reclassification of non-invasive follicular variant of papillary thyroid carcinoma as “tumors” (NIFTP), the challenge of diagnosing carcinoma lies in recognizing invasion: blood vessel invasion (BVI) or capsular invasion (CI). We aimed to assess reproducibility in diagnosing BVI and CI in follicular-patterned tumors using a web-platform, under conditions as close as possible to routine surgical pathology practice. Six-hundred-fourteen pathologists reviewed 69 thyroid lesions diagnostically challenging for BVI or CI, made available in three rounds: before, immediately after, and three months after a training lecture given by an expert pathologist. Each lesion was associated with a quiz (five queries: certain CI, doubtful CI, certain BVI, doubtful BVI, or no invasion). Intraobserver agreement was fair for five queries and moderate for two categories (i.e., BVI vs. the other four queries). Interobserver overall agreement (OA) of participants was fair for all five queries and moderate for two categories (OA for five queries: 0 at each round; OA [95% CI] for two categories: Round 1 to 3: 8.7 [3.3–18.0], 2.9 [0.4–10.1], 4.3 [0.9–12.2]). Importantly, participants agreement with expert consensus improved following the training lecture and was maintained for three months (OA for five queries: Round 1 to 3: 40.6 [13.0–62.3], 49.3 [21.7–69.6], 49.3 [21.7–78.3]; OA for two categories: Round 1 to 3: 72.5 [60.9–89.9], 76.8 [62.3–88.4], 75.4 [60.9–89.9]). This study reveals the difficulty of applying clearly defined BVI and CI criteria to routine practice, but a specifically designed training lecture improves diagnostic accuracy, and the improvement is retained over months.

**Supplementary Information:**

The online version contains supplementary material available at 10.1007/s00428-025-04045-1.

## Introduction

Encapsulated/circumscribed thyroid follicular-patterned neoplasms are very common [1]. For decades, many were diagnosed as follicular variant papillary carcinoma (FvPTC) based on the identification of papillary carcinoma-type nuclear alterations, often without clarifying whether capsular or vascular invasion were present [2]. Determining if nuclei exhibit papillary carcinoma-type characteristics has been a persistent challenge for pathologists, with extensive literature demonstrating low diagnostic reproducibility, even among thyroid pathology experts [3–6].

In 2016, non-invasive encapsulated FvPTC, accounting for 0.5–20% of thyroid carcinomas, was reclassified due to its exceptionally low recurrence risk [7]. To prevent over-diagnosis and over-treatment, these tumors are now termed “Non-Invasive Follicular Thyroid Neoplasm with Papillary-Like Nuclear Features” (NIFTP) [1, 7–9]. The presence of blood vessel invasion (BVI) or capsular invasion (CI) is now the primary criterion for diagnosing malignancy, regardless of nuclear characteristics. In essence, an encapsulated follicular-patterned tumor is considered malignant only if BVI or CI can be found.

However, the diagnosis of BVI and CI can also be problematic [1, 10–12]. Many cases with challenging BVI or CI diagnoses are referred to experts for a second opinion [13]. To the best of our knowledge, over the years, only three studies with limited cases and participants have explored the reproducibility of BVI and/or CI diagnosis in follicular-patterned thyroid nodules [13–15]. Results indicate significant variation among observers.

In this study, our first aim is to evaluate the reproducibility of the diagnosis of BVI and CI [16] in a large series of circumscribed follicular-patterned tumors under conditions as close as possible to those of daily practice — i.e., by using virtual slides of whole histology sections — and by enrolling a large number of surgical pathologists from different countries, whatever their experience. Our second aim is to determine if e-learning could improve the accuracy of BVI and CI diagnoses, and if learning could be maintained. For this purpose, we developed a novel teaching protocol, defined by the acronym MERLOT: i.e., Model to Evaluate the Real Long-term impact Of Teaching.

## Materials and Methods

### Participants

Pathologists, including both residents and experienced pathologists, were invited to participate via national pathology society mailing lists in France, other French-speaking countries, Italy, and Portugal (Supplementary Materials and Methods 1 and Supplementary Fig. 1).

### Case selection and criteria for blood vessel invasion and capsular invasion

Sixty-nine diagnostically challenging, circumscribed and well differentiated follicular-patterned tumors were selected, all with questionable BVI or CI. Nuclear alterations were not taken into consideration for the purpose of the study. Widely invasive and high-grade carcinomas were excluded.

The tumors and the areas of interest to evaluate invasion were selected by two co-authors (GB and YM) from the slide archives of the French Division of the International Academy of Pathology Annual Thyroid Paris Course and from the archives of Bordeaux University Hospital.

Dr. JK Chan criteria were followed to diagnose both BVI and CI (16) (Supplementary Materials and Methods 2 and Supplementary Fig. 2). These criteria are widely followed throughout the world and endorsed by the International Collaboration on Cancer Reporting (ICCR). According to these criteria, BVI is defined by endovascular tumor plugs protruding in the lumen, covered by endothelium or associated with fibrin, with or without attachment to the vessel wall; intratumoral or subcapsular vessels do not qualify for BVI. Following Dr. JK Chan, CI is defined by full-thickness capsular penetration, with or without a “mushroom” or newly formed thin fibrous covering of the protruding tumor (16).

### Training lecture

A virtual training lecture on BVI and CI diagnosis based on Dr. JK Chan’s criteria as outlined above was delivered via Zoom to national audiences using the same English PowerPoint presentation (Supplementary Materials and Methods 2).

### Experts

Three experts with a specific interest in thyroid pathology (GB, GT and MSS) participated in the study and gave the training lectures to their respective national audiences (Supplementary Materials and Methods 2).

### Evaluation of invasion on virtual slides

One slide of each tumor with the area of interest to evaluate BVI or CI was reviewed by experts and non-expert participants using a customized web platform.

**Fig. 1:**
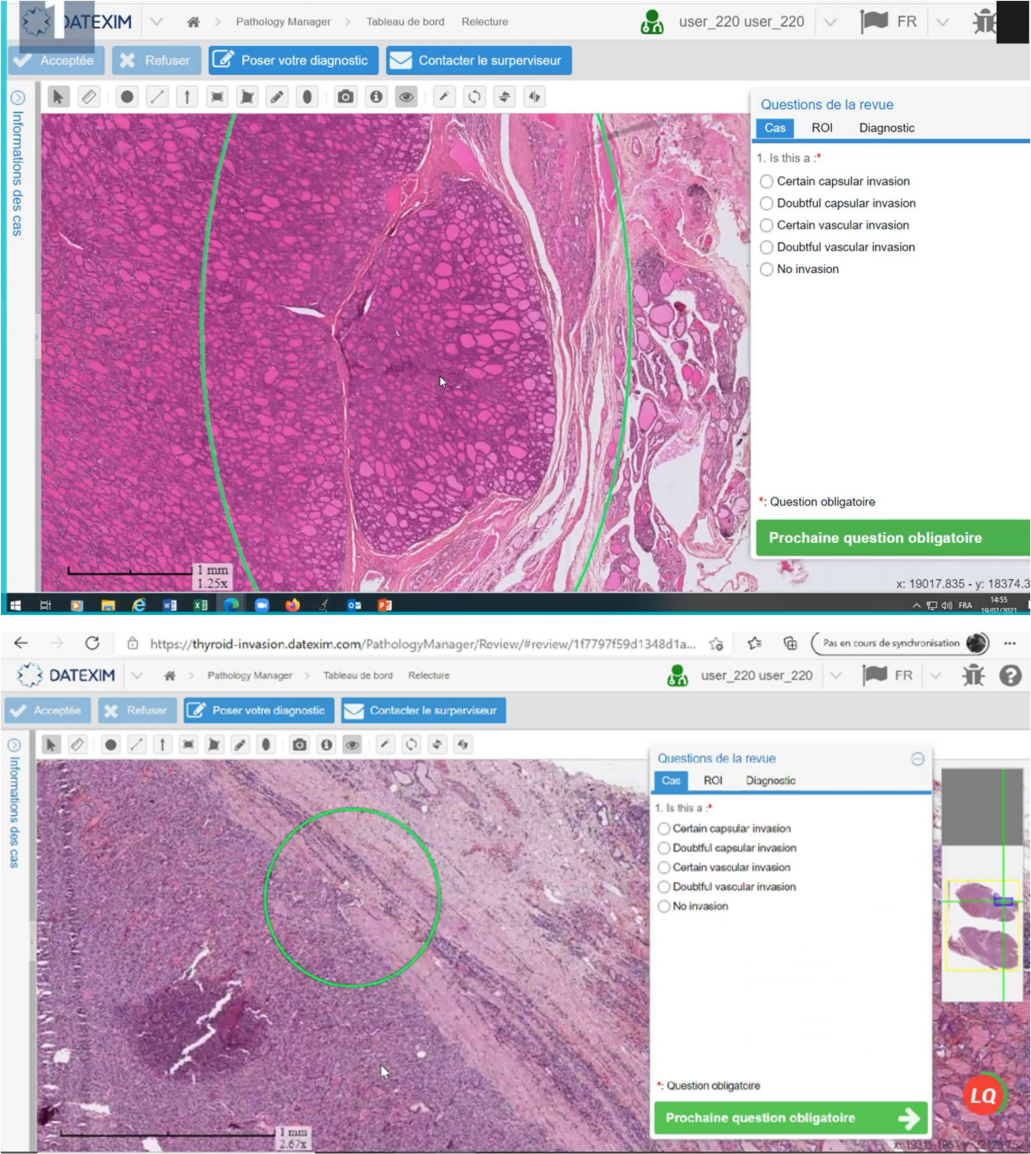
Screenshots of whole-slide images on the Datexim IMS full-web viewer, as seen by the participants to the training course. The areas submitted for analysis are marked with green circles. The same questionnaire was used for each lesion and at each round

The areas of interest (i.e., focus of possible BVI or CI) were marked with circles on all virtual slides (Supplementary Materials and Methods 3) (Fig. 1).

### e-learning MERLOT protocol

Our MERLOT teaching protocol consisted of three rounds during which the participants evaluated the possible BVI or CI lesions (Supplementary Materials and Methods 4).

- Round 1: evaluation of the lesions before the training lecture.
- Round 2: re-evaluation of the lesions shortly after the training lecture to assess short-term learning.
- Round 3: re-evaluation of the lesions approximately 3 months after the training lecture to assess long-term learning.

Participants used the same questionnaire for each lesion, selecting from: “Certain capsular invasion (CI)/Doubtful CI/Certain vascular invasion (VI)/Doubtful VI/No invasion”. Lesion presentation order varied in each round, and responses were recorded anonymously.

The three experts analyzed the 69 lesions in two rounds, 6 months apart, using the same questionnaire and anonymous answers as the participants. Expert consensus was reached for each lesion after three online meetings and was used as the gold standard for the purpose of the study.

### Statistical analysis

Data were analyzed according to all five queries presented in the questionnaire (certain CI, doubtful CI, certain BVI, doubtful BVI or no invasion), as well as according to only two categories, i.e., certain BVI only vs. the four other queries combined (certain CI, Doubtful CI, Doubtful BVI and no invasion).

Established procedures were followed (Supplementary Materials and Methods 5). Agreement was assessed using overall agreement (OA) and the non-weighted kappa coefficient (K). OA measures the proportion of similar readings among participants, while K considers chance agreement. Interpretation of K followed the conventional scale shown in Supplementary Table 1.

Inter- and intraobserver reproducibility was analyzed for the three experts (Supplementary Table 2) and the non-expert participants. Agreement of both expert and non-expert participants with the expert consensus was also assessed (Supplementary Materials and Methods 5).

## Results

A total of 614 participants analyzed at least one lesion in the study. Among them, 211 participants scored all 69 lesions in all three rounds, they were the core object of our statistical analyses. The 252 participants who answered at least 80% of the lesions for rounds 1, 2, and 3 were the object of subgroup analyses (Supplementary Table 3). Out of these 252 participants, 218 completed the survey on their professional profile (Supplementary Table 4). Table 1 provides information about participants.

**Table 1:** Demographics of participant pathologists

Type of practice (N=218)	
Resident	84 (38.5%)
University Hospital or Cancer Center	65 (29.8%)
General Hospital	40 (18.3%)
Private practice	27 (12.4%)
Retired	2 (0.9%)
**Trained pathologists (excluding residents) (N=134)**	
***Years of practice***	
Median (Range)	17.5 (1.0: 44.0)
***Consultation Cases received***	
No	105 (78.4%)
Yes	29 (21.6%)
***Approximate number of thyroid gland surgical cases diagnosed per year***	
Median (Range)	42.5 (0.0:1000)

**Fig. 2:**
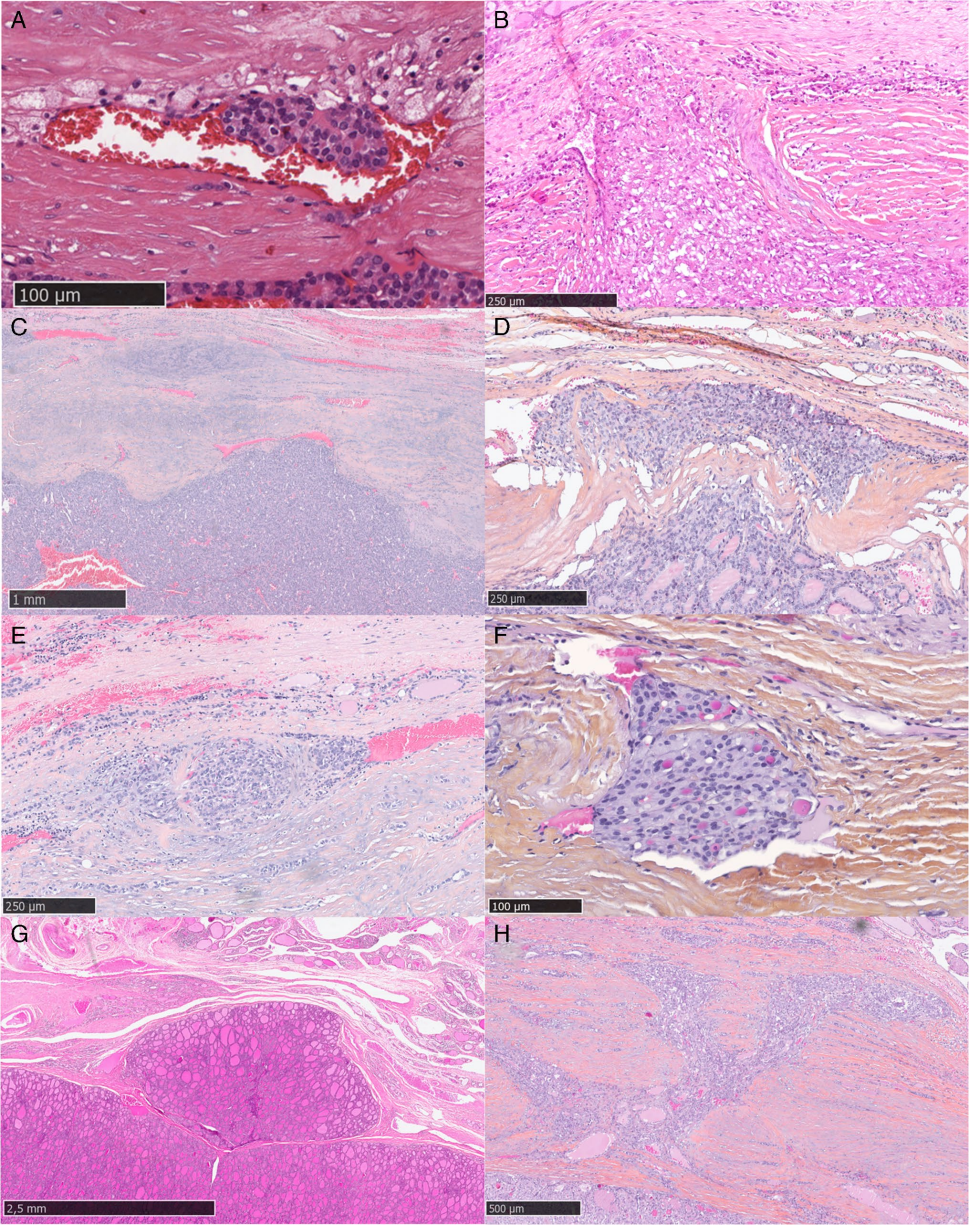
Lesion (n. 16) with agreement among experts: expert consensus certain blood vessel invasion (**A**). Lesion (n. 81) with agreement among experts: expert consensus certain capsular invasion (**B**). Lesion (n. 74) with agreement among experts: expert consensus no invasion (**C**). Lesion (n. 59) without agreement among experts: expert consensus doubtful vascular invasion (**D**). Lesion (n. 76) without agreement among experts: expert consensus doubtful vascular invasion (**E**). Lesion (n. 68) without agreement among experts: expert consensus doubtful vascular invasion (**F**). Lesion (n. 53) without agreement among experts: expert consensus doubtful capsular invasion (**G**). Lesion (n. 69) without agreement among experts: expert consensus no invasion (**H**)

Considering all five queries in the questionnaire (certain CI, doubtful CI, certain BVI, doubtful BVI, or no invasion), the expert consensus categorized 26/69 lesions (37.7%) as “certain BVI,” 13/69 lesions (18.8%) as “doubtful BVI,” 5/69 lesions (7.2%) as “certain CI,” 2/69 lesions (2.9%) as “doubtful CI,” and 23/69 lesions (33.3%) as “no invasion” (Fig. 2) (Supplementary Table 5).

### Expert results

**Fig. 3:**
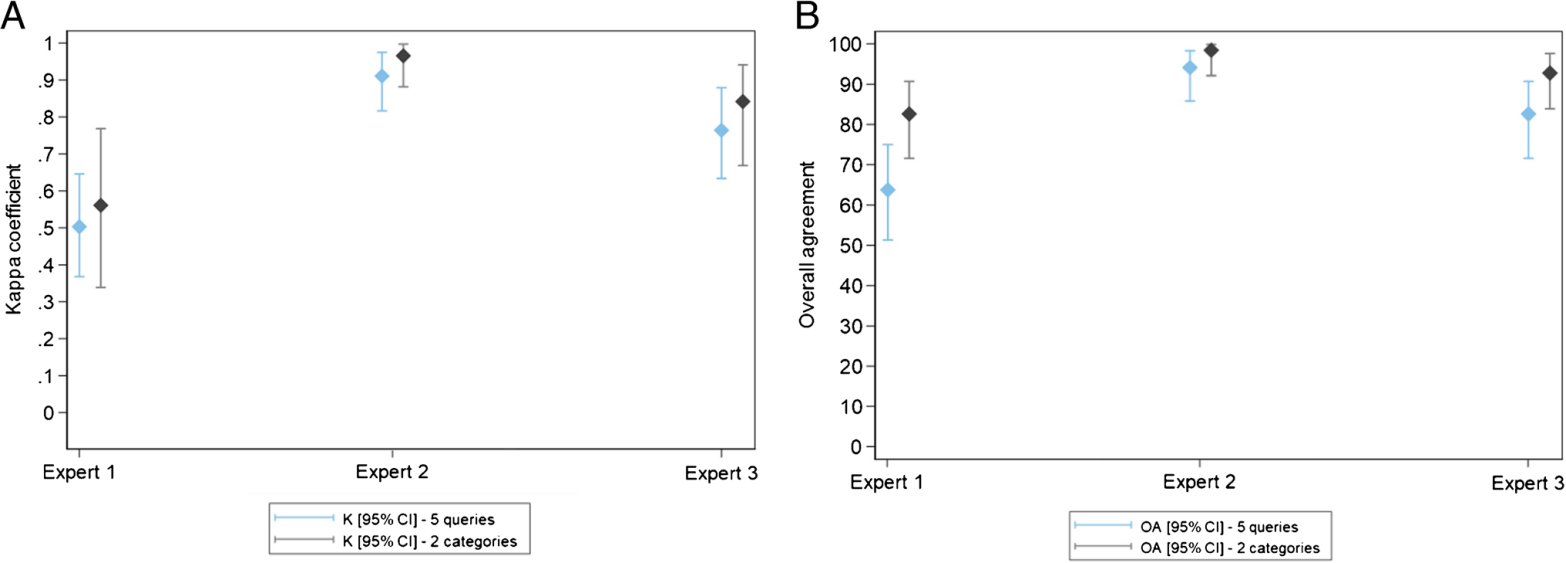
Expert results—Intraexpert agreement on the queries presented in the questionnaire. Box plot diagrams: Kappa coefficient (**A**) and overall agreement (**B**) for each expert according to the number of queries (five queries and two categories, BVI vs. the other 4 queries). Blue for all five queries and black for two categories (BVI vs. the other 4 queries). The results (K and OA) are presented with their confidence interval at 95%

For all five queries and when considering only two categories (certain BVI vs. the 4 other queries), *intraexpert agreement* was moderate to almost perfect for the three experts (Table 2; Fig. 3).

**Table 2:** Expert results - Intraexpert and interexpert agreement on the queries presented in the questionnaire and agreement of each expert with the expert consensus

Three experts: 69/69 lesions scored after expert rounds 1 and 2				All five queries	Two categories (BVI vs. the other four queries)
Intraexpert agreement	Expert 1		K [95% CI]	0.503 [0.368: 0.646]	0.561 [0.339: 0.768]
			OA (%) [95% CI]	63.8 [51.3: 75.0]	82.6 [71.6: 90.7]
	Expert 2		K [95% CI]	0.914 [0.819: 0.978]	0.968 [0.884: 1.000]
			OA (%) [95% CI]	94.2 [85.8: 98.4]	98.6 [92.2: 100.0]
	Expert 3		K [95% CI]	0.764 [0.634: 0.879]	0.842 [0.668: 0.941]
			OA (%) [95% CI]	82.6 [71.6: 90.7]	92.8 [83.9: 97.6]
Interexpert agreement	Round 1		K [95% CI]	0.328 [0.23: 0.449]	0.553 [0.410: 0.711]
			OA [95% CI]	37.7 [26.3: 50.2]	69.6 [57.3: 80.1]
	Round 2		K [95% CI]	0.372 [0.260: 0.494]	0.539 [0.377: 0.706]
			OA [95% CI]	40.6 [28.9: 53.1]	71 [58.8: 81.3]
Agreement with expert consensus	Round 1	Expert 1	K [95% CI]	0.41 [0.273: 0.555]	0.526 [0.316: 0.736]
			OA [95% CI]	58 [45.5: 69.8]	78.3 [66.7: 87.3]
		Expert 2	K [95% CI]	0.589 [0.450: 0.721]	0.782 [0.606: 0.911]
			OA [95% CI]	71 [58.8: 81.3]	89.9 [80.2: 95.8]
		Expert 3	K [95% CI]	0.562 [0.426: 0.701]	0.687 [0.496: 0.853]
			OA [95% CI]	68.1 [55.8: 78.8]	85.5 [75.0: 92.8]
	Round 2	Expert 1	K [95% CI]	0.543 [0.397: 0.688]	0.486 [0.290: 0.730]
			OA [95% CI]	66.7 [54.3: 77.6]	78.3 [66.7: 87.3]
		Expert 2	K [95% CI]	0.608 [0.474: 0.744]	0.749 [0.574: 0.883]
			OA [95% CI]	72.5 [60.4: 82.5]	88.4 [78.4: 94.9]
		Expert 3	K [95% CI]	0.585 [0.446: 0.728]	0.72 [0.547: 0.878]
			OA [95% CI]	69.6[57.3: 80.1]	87 [76.7: 93.9]

**Fig. 4:**
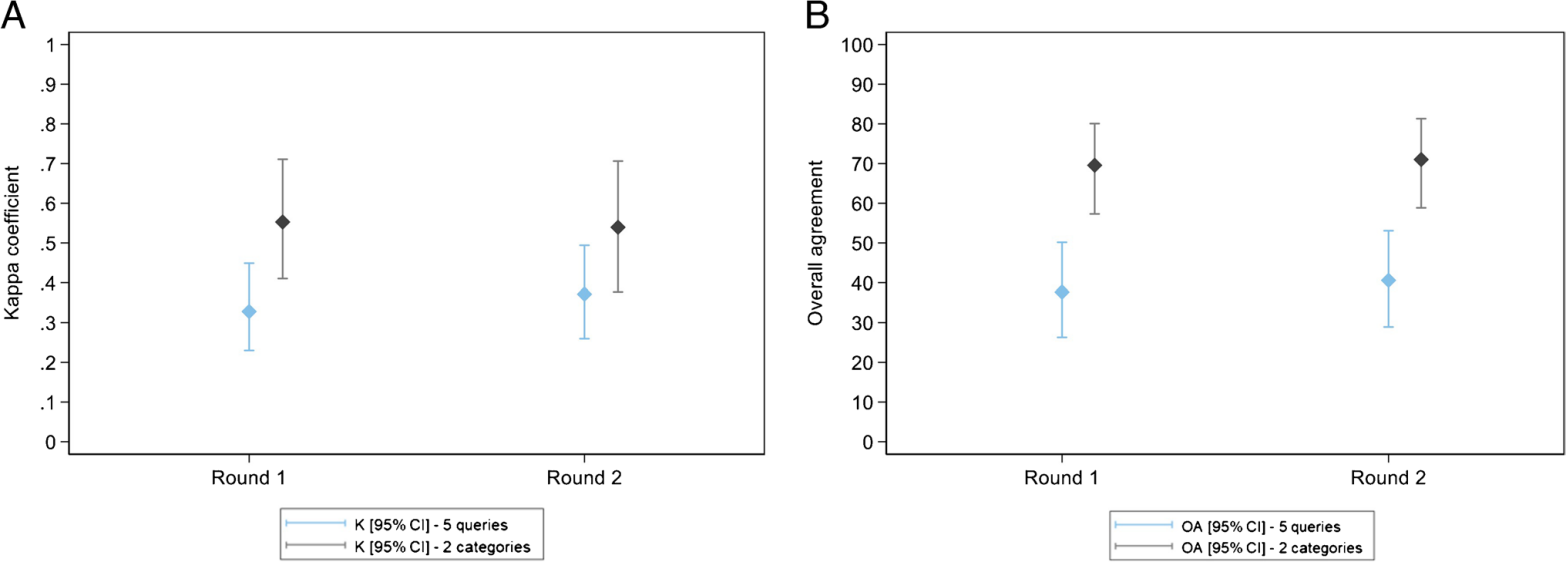
Expert results—Interexpert agreement on the queries presented in the questionnaire. Box plot diagrams: Kappa coefficient (**A**) and overall agreement (**B**) according to the rounds. Blue for all five queries and black for two categories (BVI vs. the other 4 queries). The results (K and OA) are presented with their confidence interval at 95%

**Fig. 5:**
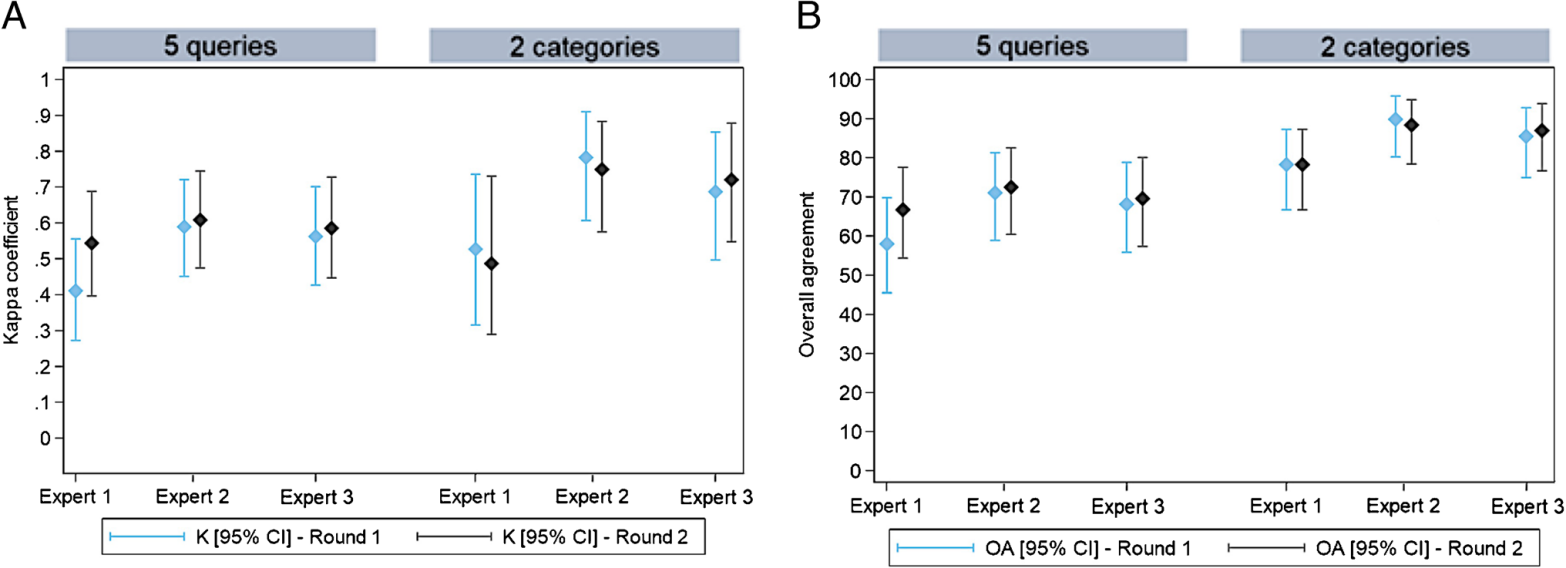
Expert results—Agreement of each expert with the expert consensus. Box plot diagrams. Kappa coefficient (**A**) and overall agreement (**B**) for each expert according to the number of queries. Blue for round 1 and black for round 2. The results (K and OA) are presented with their confidence interval at 95%

*Interexpert agreement* was fair to moderate (Table 2; Fig. 4). For each expert, *agreement with the expert consensus* was moderate to substantial and comparable for all five queries or only two categories (certain BVI vs. the other four queries) (Table 2; Fig. 5).

### Participant results

#### Intraobserver agreement

**Fig. 6:**
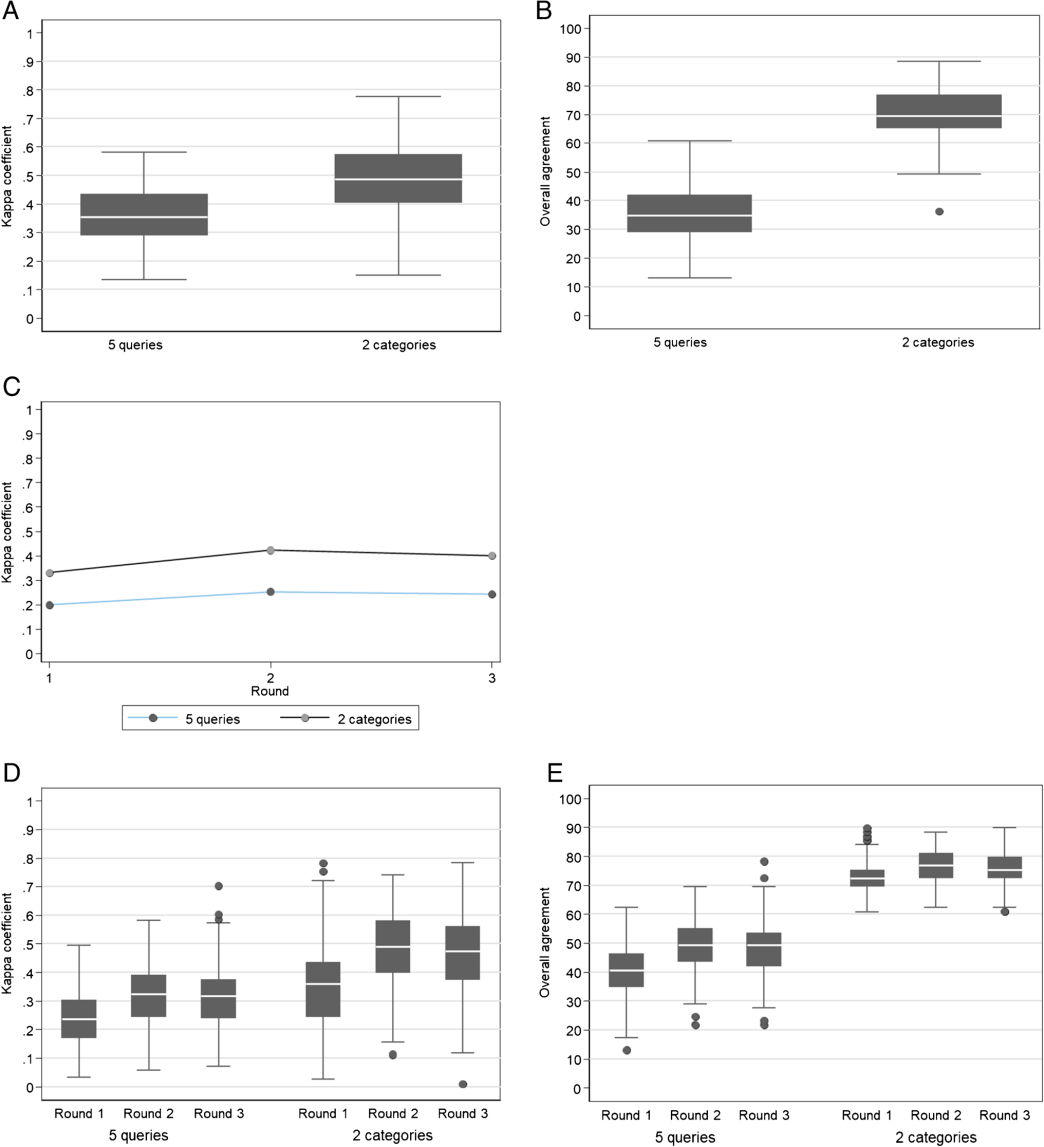
Participant results, rounds 1, 2 and 3, participants with 100% of lesions scored (211 participants). **A**–**B** Intraobserver agreement among the participants for all five queries and two categories only (BVI vs. the other 4 queries): Kappa coefficient (**A**) and overall agreement (**B**) according to the number of queries. The bottom/top of the box represents the first (Q1)/third (Q3) quartiles, the bold line inside the box represents the median and the two bars outside the box represent the lowest/highest value within 1.5 × the inter-quartile range. Outliers are represented with circles. **C** Interobserver agreement among participants: evolution of the kappa coefficient according to the rounds. Blue for all five queries, black for two categories (BVI vs. the other 4 queries). **D**–**E** Agreement with the expert consensus: Kappa coefficient (**D**) and overall agreement (**E**) according to the number of queries (five queries and two categories, BVI vs. the other 4 queries) and rounds. The bottom/top of the box represents the first (Q1)/third (Q3) quartiles, the bold line inside the box represents the median and the two bars outside the box represent the lowest/highest value still within 1.5 × the inter-quartile range. Outliers are represented with circles

Table 3 shows fair intraobserver agreement for all five queries among participants who scored 69/69 lesions for three rounds (median kappa = 0.356). For two categories (certain BVI vs. other four queries), agreement was moderate (median kappa = 0.486) (Fig. 6A–B).

**Table 3:** Participant results - Intraobserver and interobserver agreement among the participants and agreement with the expert consensus for all five queries and two categories only (BVI vs. the other four queries)

211 participants with 69/69 lesions scored at rounds 1, 2 & 3			All five queries	Two categories (BVI vs. the other four queries)
Intraobserver agreement		K median [range]	0.356 [0.134: 0.580]	0.486 [0.149: 0.776]
		OA (%) median [range]	34.8 [13.0: 60.9]	69.6 [36.2: 88.4]
Interobserver agreement	Round 1	Combined K value	0.1994	0.330
		OA [95% CI] (%)	0	8.7 [3.3: 18.0]
	Round 2	Combined K value	0.2526	0.423
		OA [95% CI] (%)	0	2.9 [0.4: 10.1]
	Round 3	Combined K value	0.2435	0.400
		OA [95% CI] (%)	0	4.3 [0.9: 12.2]
Agreement with expert consensus	Round 1	K median [range]	0.236 [0.034: 0.494]	0.36 [0.026: 0.782]
		OA (%) median [range]	40.6 [13.0: 62.3]	72.5 [60.9: 89.9]
	Round 2	K median [range]	0.324 [0.058: 0.581]	0.491 [0.111: 0.741]
		OA (%) median [range]	49.3 [21.7: 69.6]	76.8 [62.3: 88.4]
	Round 3	K median [range]	0.317 [0.071: 0.702]	0.474 [0.009: 0.782]
		OA (%) median [range]	49.3 [21.7: 78.3]	75.4 [60.9: 89.9]

#### Interobserver agreement

Considering all five queries, the combined kappa was slight for round 1 (combined kappa = 0.1994). Improvement occurred after a training lecture for round 2 and was maintained for round 3 (combined kappa = 0.2526 in round 2 and 0.2435 in round 3). None of the 211 participants scored the same set of queries for the 69 lesions in any of the three rounds (OA = 0%) (Table 3).

For two categories (certain BVI vs. the other four queries), the combined kappa for interobserver agreement was fair for round 1 (combined kappa = 0.330). The kappa increased to moderate after the training lecture (combined kappa = 0.423 for round 2 and 0.400 for round 3). Among 211 participants, there were instances of agreement for round 1 (OA = 8.7%), round 2 (OA = 2.9%), and round 3 (OA = 4.3%) (Table 3; Fig. 6C).

For each individual query, interobserver agreement was fair for certain CI and certain BVI at rounds 1 and 3. It was moderate for certain BVI at round 2, slight to fair for no invasion, and slight for doubtful CI and doubtful BVI (Supplementary Table 6).

#### Agreement of the participants with expert consensus

For all five queries, the median kappa was fair with the expert consensus for all three rounds. However, agreement improved after the training lecture and remained improved long-term. Median kappa coefficients were as follows: 0.236 (range: 0.034–0.494) for round 1; 0.324 (range: 0.058–0.581) for round 2; 0.317 (range: 0.071–0.70) for round 3. For two categories (certain BVI vs. other four queries), agreement improved from fair to moderate after the training lecture. Median kappa coefficients were as follows: 0.360 (range: 0.026–0.782) for round 1; 0.491 (range: 0.111–0.741) for round 2; 0.474 (range: 0.009–0.782) for round 3 (Table 3; Fig. 6D–E).

#### Evolution of the kappa coefficient and overall agreement of the participants with the expert consensus between rounds

The training lecture significantly improved kappa coefficients between rounds 1 and 2 for all five queries and considering only two categories (certain BVI vs. other four queries).

For all five queries, 80.6% of participants improved K coefficients between rounds 1 and 2. Between rounds 1 and 3, 77.3% improved, and between rounds 2 and 3, 43.1% improved (Supplementary Table 7). When considering only two categories (certain BVI vs. other four queries), the trend was similar. 78.7% improved between rounds 1 and 2, 79.1% between rounds 1 and 3, and 41.7% between rounds 2 and 3 (Supplementary Table 7).

### Subgroup analysis

Subgroup analyses were performed with data from the 252 participants that scored at least 80% of the lesions at rounds 1, 2 and 3.

#### Lesions with highest consensus among experts and participants

The 69 cases in this study were diagnostically challenging for BVI or CI. Thirty lesions were particularly difficult: consensus among experts was low (agreement was < 4/8, Supplementary Table 2) and agreement of participants with the expert consensus was only slight (Supplementary Table 8). Exclusion of these 30 lesions led to improvements in both Kappa coefficients and overall agreement for the remaining 39 lesions, among both participants (Supplementary Table 9) and experts (Supplementary Table 10).

#### Pathology expertise, country of practice and professional context

Trained pathologists showed significantly better agreement with expert consensus than residents, in all three rounds for all five queries. When considering only two categories (certain BVI vs. the other four queries), trained pathologists significantly outperformed residents only in rounds 2 and 3 (Supplementary Table 11). There were no significant differences between French and French-speaking country participants, Italian participants, and Portuguese participants (Supplementary Table 11).

Similarly, agreement with expert consensus showed no significant differences when considering the type of professional setting (University Hospital/Cancer Center, General Hospital, private practice, Supplementary Table 12), annual number of thyroid cases signed out (Supplementary Table 13), and years in surgical pathology practice (Supplementary Table 14). Agreement with expert consensus did not significantly differ between trained pathologists who routinely received thyroid lesions for a second opinion and those who did not (data not shown).

## Discussion

Malignant, encapsulated, follicular-patterned tumors are histologically distinguished from benign ones by BVI or CI. Diagnosing invasion can be very challenging [1, 17–20]. To diagnose BVI and CI, we followed the widely accepted criteria outlined by Dr. JK Chan in 2007 [16].

In spite of expert consensus being reached for all lesions, this study highlights the substantial difficulties associated in diagnosing both BVI and CI. Among participants, inter- and intraobserver variability exhibited disappointingly low levels of agreement. We believe these disappointing levels are not due to the ambiguity of Dr. JK Chan’s criteria, but rather to the inherent complexity of some real-life cases like those selected for our study.

Considering only two categories (certain BVI vs. the other four queries) — instead of all five in the questionnaire (certain CI, doubtful CI, certain BVI, doubtful BVI, or no invasion), agreement with consensus improved**.** The analysis of BVI vs. the other four other queries combined was performed because certain BVI is considered the most relevant criterion for malignancy in follicular-patterned tumors, with prognostic — in addition to diagnostic — implications (1). Only five cases had certain CI without BVI (certain or questionable), and statistical analysis of certain CI and certain BVI vs. the three remaining queries did not substantially change the results of the study (data not shown).

This indicates that pathologists can often distinguish between benign (adenoma) — or almost benign (NIFTP and tumor of uncertain malignant potential) — diagnoses, and a definitive diagnosis of carcinoma.

Difficulties in diagnosing invasion in encapsulated follicular-patterned tumors have been the topic of very few studies. Only three addressed its reproducibility. In 2002, Hirokawa et al. showed diagnostic disagreement among eight experts in the distinction between FA and FTC, in a small series of twenty-one difficult lesions without reproducibility analysis [14]. Franc et al. demonstrated in a series of forty-one cases reviewed by five pathologists that diagnosing BVI and CI lacks reproducibility [13]. In 2020, Zhu et al. showed low reproducibility in the diagnosis of CI on twenty still images analyzed by eleven Asian pathology experts [15].

Our study is the first to evaluate the reproducibility of CI and BVI diagnoses in follicular-patterned tumors while also assessing the effectiveness of teaching using pre-defined and widely accepted criteria. Interestingly, country of practice, educational background, and professional context did not significantly influence participant results.

Few scientific publications have examined e-learning efficacy in histopathology, with fewer than 30 in the past decade [21]. Our study reveals how a well-planned training lecture can improve the diagnosis of invasion in encapsulated follicular-patterned tumors, and that learning can be retained months after the initial training lecture.

Overall, the results of the e-learning MERLOT course project are fully compatible with the Ebbinghaus forgetting curve [22], confirming the need to repeat the same training process for the information to be retained. Thus, MERLOT can potentially be easily applied to improve diagnostic competence in surgical pathology as well as in other medical fields.

Our study shows that trained pathologists performed better than residents in training after the teaching lecture. This is probably due to their prior knowledge, experience and judgement, that enable them to absorb new information more thoroughly.

This study has limitations. We deliberately chose challenging cases, with a majority involving BVI, as it is a crucial criterion to diagnose malignancy in encapsulated follicular-patterned tumors. Exclusion of the most difficult cases improved agreement and reproduced conditions probably closer to the daily practice, as most routine cases are simpler to deal with than those selected for our study.

Moreover, our gold standard consensus relied on the opinion of thyroid pathology experts. Ideally, validating diagnoses and criteria should involve long clinical follow-up (recurrence vs. no recurrence). However, this is challenging due to the slow evolution of well-differentiated follicular-patterned tumors [23]. A comprehensive, long-term study is needed to establish gold standards, as previously done for nuclear papillary carcinoma-type alterations [7].

Importantly, thyroid cancer may be a subject of litigation [8, 24]. Given the low reproducibility in diagnosing invasion, this study may support pathologists facing malpractice claims [25].

To summarize, this study demonstrates that to diagnose capsular and blood vessel invasion in follicular-patterned lesions is difficult. However, our MERLOT e-learning protocol shows that pathologists can improve their performance after a training lecture with a positive long-term impact of at least three months.

## Supplementary Information

Below is the link to the electronic supplementary material.

Supplementary File 1 (DOCX 834 KB)

Supplementary Material and Methods (DOCX 21.2 KB)

Supplementary Tables (DOCX 57.7 KB)

## Data Availability

The datasets generated during and/or analyzed during the current study are available from the corresponding author on reasonable request.
