# Supplementary material for: Can we improve the diagnosis of invasion in encapsulated follicular-patterned thyroid tumors? Data from a massive international e-learning initiative
Source: Virchows Arch. 2025 Feb 24;487(1):105–16. doi: 10.1007/s00428-025-04045-1 (PMC12289735; doi:10.1007/s00428-025-04045-1)
Supplement: Supplementary file 1 — (DOCX 834 KB) [file 428_2025_4045_MOESM1_ESM.docx]

******

**Supplemental Figure 1.** Screenshot of the questionnaire sent to the participants to obtain information on their professional background, with the information requested at the link [Who are you? (google.com)](https://docs.google.com/forms/d/e/1FAIpQLSeGzJ27r50sbigdWtQ4dv_yIv7vNEMQJpXHcFXgmPmhR66tjg/viewform)


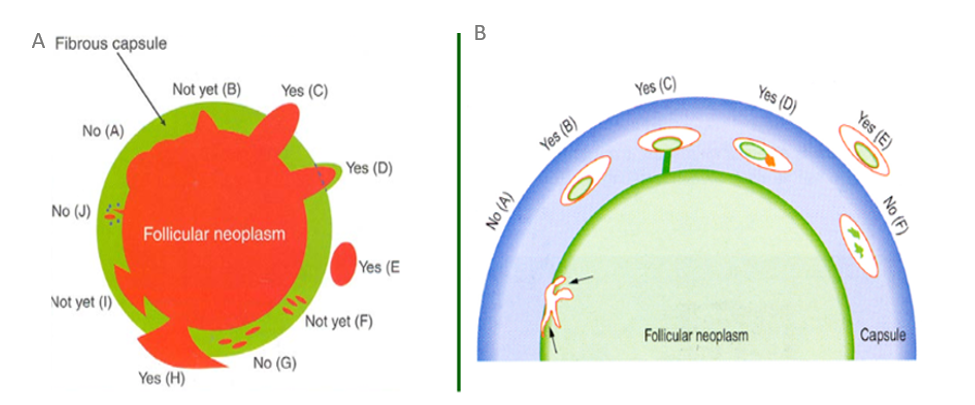


**Supplemental Figure 2. Criteria of capsular invasion and blood vessel invasion according to Dr. JK Chan** [Chan JK (2007). Tumors of the thyroid and parathyroid glands. In: Diagnostic Histopathology of tumors. Fletcher CDM. Churchill Livingstone Elsevier, Philadelphia]

A. Criteria for capsular invasion:

Schematic drawing for the interpretation of the presence or absence of CI. The diagram depicts a follicular neoplasm (orange) surrounded by a fibrous capsule (green). a) bosselation on the inner aspect of the capsule does not represent CI; b) sharp tumor bud invades into but not through the capsule suggesting invasion requiring deeper sections to exclude; c) tumor totally transgresses the capsule invading beyond the outer contour of the capsule qualifying as CI; d) tumor clothed by thin (probably new) fibrous capsule but already extending beyond an imaginary (dotted) line drawn through the outer contour of the capsule qualifying as CI; e) satellite tumor nodule with similar features (architecture, cytomorphology) to the main tumor lying outside the capsule qualifying as CI; f) Follicles aligned perpendicular to the capsule suggesting invasion requiring deeper sections to exclude g) follicles aligned parallel to the capsule do not represent CI; h) mushroom-shaped tumor with total transgression of the capsule qualifies as CI; i) mushroom-shaped tumor within but not through the capsule suggests invasion requiring deeper sections to exclude; j) neoplastic follicles in the fibrous capsule with a degenerated appearance accompanied by lymphocytes and siderophages does not represent CI but rather capsular rupture related to prior fine needle aspiration.

B. Criteria for blood vessel invasion:

The diagram depicts a follicular neoplasm (green) surrounded by a fibrous capsule (blue). a) Bulging of tumor into vessels within the tumor proper does not constitute VI. b) Tumor thrombus covered by endothelial cells in intracapsular vessel qualifies as VI. c) Tumor thrombus in intracapsular vessel considered as VI since it is attached to the vessel wall. d) Although not endothelialized, this tumor thrombus qualifies for VI because it is accompanied by a fibrin thrombus. e) Endothelialized tumor thrombus in vessel outside the tumor capsule represents VI. f) Artefactual dislodgement of tumor manifesting as irregular tumor fragments into vascular lumen unaccompanied by endothelial covering or fibrin thrombus.
