# Supplementary material for: Can we improve the diagnosis of invasion in encapsulated follicular-patterned thyroid tumors? Data from a massive international e-learning initiative
Source: Virchows Arch. 2025 Feb 24;487(1):105–16. doi: 10.1007/s00428-025-04045-1 (PMC12289735; doi:10.1007/s00428-025-04045-1)
Supplement: Supplementary file 2 — Supplementary Material and Methods (DOCX 21.2 KB) [file 428_2025_4045_MOESM2_ESM.docx]

**SUPPLEMENTAL INFORMATION**

**Supplemental Material and Methods**

**1 – Participants - Professional details.** Participants provided professional details: trained pathologists or pathology residents, years of practice, current residence and practice country, additional board certifications, number of thyroid surgical pathology cases signed out per year, and whether or not the participant routinely receives thyroid lesions for second opinion.

**2 -** **CI and BVI definitions and virtual training lecture.** Malignant, encapsulated, follicular-patterned tumors are histologically distinguished from benign ones by CI and/or BVI. The WHO 5th edition defines CI as "full-thickness capsular penetration", often with a mushroom-shaped growth (1). Some experts accept partial capsule penetration for a carcinoma diagnosis (11), while most pathologists - following Dr. JK Chan's criteria - require complete transgression of the capsule, with or without a newly formed thin fibrous covering or a tumor cell “mushroom” (16).

Diagnosing BVI in follicular-patterned thyroid tumors is challenging and more clinically relevant than CI (1,17–20). The WHO defines BVI as “invasion of vessels within the tumor capsule or beyond, with intravascular tumor attached to the vessel wall, or admixed with fibrin or covered by endothelium” (1). Most pathologists follow Dr. JK Chan criteria for BVI: tumor plugs (protruding, polypoid in shape) have to be covered by endothelium or associated with fibrin thrombus, attached to the vessel wall or not, in blood vessel inside or outside (but not inside) the tumor capsule (16).

In our study, Dr. JK Chan's criteria served as the diagnostic standard for CI and BVI, and the virtual training lecture on BVI and CI was based on them (**Supplemental Figure 2**).

The training lecture was a Zoom videoconference held in French by GB, in Italian by GT and in Portuguese by MSS to their respective French- speaking, Italian-speaking and Portuguese-speaking audiences. All lecturers used exactly the same power-point presentation written in English. The lesions shown to the participants during the training lecture were different from those submitted to the them for the test rounds.

**3 - Evaluation of invasion on virtual slides.** All selected slides were scanned at 40X magnification and converted into whole-slide images using the Nanozoomer 2.0-HT c10730-12 platform from Hamamatsu, Japan. These images were accessible through the Datexim IMS web platform, integrated into a remote diagnosis website customized for the study. The areas of interest for the evaluation of invasion (*i.e.,* foci of possible BVI or CI) were marked with circles on all virtual slides (**Figure 1**). To avoid confusion, if a slide had several of these lesions, the virtual slide was duplicated as many times as there were lesions. The present study used virtual slides allowing for a large number of cases to be analyzed at different times before and after the training lecture. Virtual slides of whole sections are closer to daily practice than still images, focused only on one part of a histological section. Indeed, virtual slides allow for the examination of a specific lesion in its *real* context, at different magnifications, as in the case of real-life pathology practice.

**4 -** **e-learning MERLOT protocol.** The e-learning MERLOT (Model to Evaluate the Real Long-term impact Of Teaching) protocol is a spin-off of the Annual Thyroid Paris Course. This 2-day course consists of lectures alternating with discussions of cases for practising pathologists and has been given on behalf of the French Division of the International Academy of Pathology since the late 1990s. In June 2020, the course could not take place in person due to the COVID epidemic. Under the leadership of one of the authors (GB), the cancellation of the course was transformed into an opportunity to develop a novel teaching method: the MERLOT protocol. With this protocol the teaching was expanded to over 600 participants from various countries, one-third of whom scored all lesions in all three rounds (see Results section). Participants received no feedback on their scores for the 69 lesions in any of the rounds till the course was completed.

**5 - Statistical analysis.** Data were analyzed taking into account two items:

(i) Answers provided: according to all five queries presented in the questionnaire (certain CI, doubtful CI, certain BVI, doubtful BVI or no invasion) as well as according to only two categories, *i.e.,* certain BVI only vs. all the other queries combined (certain CI, Doubtful CI, Doubtful BVI and no invasion). This scheme was tested because certain BVI is the most important criterion for malignancy in follicular-patterned tumors, with prognostic - in addition to diagnostic - implications (1).

(ii) According to the number of lesions analyzed by the participants at each examination round. We conducted analyses with participants who scored all lesions (69/69 lesions) in rounds 1, 2, and 3. Additionally, to increase data power, we considered responses from participants who scored at least 80% of the lesions in rounds 1, 2, and 3, to conduct subgroup analyses.

Data were comprehensively analyzed using various methods. Descriptive statistics were applied, including frequency and percentage for qualitative variables, and median, minimum, and maximum for quantitative variables. Agreement was assessed using overall agreement (OA) and the non-weighted kappa coefficient (K). OA measures the proportion of similar readings among participants, while K considers chance agreement. Interpretation of K followed a conventional scale. Interpretation of the kappa coefficient followed the conventional scale **(Supplemental Table 1).**

In order to evaluate inter- and intraobserver reproducibility, the three experts were asked to analyze the 69 lesions in two rounds, 6 months apart, in exactly the same way as the participants (same questionnaire, anonymous answers, order of the lesions changed between the two rounds). A consensus among experts was reached for each lesion after three online discussion meetings **(Supplemental Table 2)**. The expert consensus result was considered as the gold standard for the purpose of the study.

Intra- and interobserver agreement among the non-expert participants was also analyzed. For the agreement with the expert consensus result (gold standard), agreement of each participant with the expert consensus diagnosis was assessed for each round and results are presented with median, minimum and maximum values. The difference of the agreement with the expert consensus (OA or kappa) between two rounds was evaluated for each participant. The level of agreement was considered as increased if the difference was > 0, decreased if the difference was < 0, stable if the difference was zero.

Subgroup analysis compared participants with shared characteristics, using the Kruskal-Wallis Test for continuous variables. Statistical significance was set at p < 0.05. STATA software version 16 (StataCorp LLC, College Station, TX) was used for all statistical analyses.
