# Supplementary material for: Can we improve the diagnosis of invasion in encapsulated follicular-patterned thyroid tumors? Data from a massive international e-learning initiative
Source: Virchows Arch. 2025 Feb 24;487(1):105–16. doi: 10.1007/s00428-025-04045-1 (PMC12289735; doi:10.1007/s00428-025-04045-1)
Supplement: Supplementary file 3 — Supplementary Tables (DOCX 57.7 KB) [file 428_2025_4045_MOESM3_ESM.docx]

**Supplemental Table 1. Interpretation of the kappa coefficient**.

| **Kappa** | **Agreement** |
| --- | --- |
| 0.01-0.20 | Slight agreement |
| 0.21-0.40 | Fair agreement |
| 0.41-0.60 | Moderate agreement |
| 0.61-0.80 | Substantial agreement |
| 0.81-0.99 | Almost perfect agreement |

From Landis JR, Koch GG. The Measurement of Observer Agreement for Categorical Data. Biometrics. 1977 Mar;33(1):159

**Supplemental Table 2. Number of lesions according to agreement among the three experts at Expert rounds 1 and 2, sixth months apart.**

| **Number of lesions** | **Agreement between the three experts during rounds 1 and 2^a^** |
| --- | --- |
| 6 | 8/8 |
| 8 | 7/8 |
| 13 | 6/8 |
| 12 | 5/8 |
| 17 | 4/8 |
| 10 | 3/8 |
| 3 | 2/8 |

^a^Since there are three experts and two rounds, the proportion of agreement is counted out of eight possibilities.

**Supplemental Table 3. Number of participants per examination round according to the number of lesions scored.**

|  | 0 lesion scored | > 1 lesion scored | > 80% of lesions scored (> 56/69) | 100% of lesions scored |
| --- | --- | --- | --- | --- |
| Round 1 | 76 | 538 | 458 | 440 |
| Round 2 | 168 | 446 | 391 | 357 |
| Round 3 | 219 | 395 | 359 | 333 |
| Round 1+3 |  |  | 297 | 263 |
| Round 1+2+3 |  |  | 252 | 211 |

**Supplemental Table 4. Demographics of pathologists according to their country and type of practice.**

|  |  |  | **Country of practice (n=218)** | | | | | |
| --- | --- | --- | --- | --- | --- | --- | --- | --- |
|  | | | France | Italy | Portugal | Morocco | Tunisia | Other  countries |
|  |  |  | (N = 58) | (N = 75) | (N = 28) | (N = 18) | (N = 18) | (N = 21) |
| **Country of training (n=218)** | Algeria | | 1 (1.7%) | 0 (0.0%) | 0 (0.0%) | 0 (0.0%) | 0 (0.0%) | 1 (4.8%) |
|  | Belgium | | 0 (0.0%) | 0 (0.0%) | 0 (0.0%) | 0 (0.0%) | 0 (0.0%) | 4 (19.0%) |
|  | Cameroun | | 0 (0.0%) | 0 (0.0%) | 0 (0.0%) | 0 (0.0%) | 0 (0.0%) | 1 (4.8%) |
|  | Canada | | 0 (0.0%) | 0 (0.0%) | 0 (0.0%) | 0 (0.0%) | 0 (0.0%) | 2 (9.5%) |
|  | France | | 56 (96.6%) | 0 (0.0%) | 0 (0.0%) | 1 (5.6%) | 0 (0.0%) | 3 (14.3%) |
|  | Italy | | 0 (0.0%) | 75 (100.0%) | 0 (0.0%) | 0 (0.0%) | 0 (0.0%) | 1 (4.8%) |
|  | Madagascar | | 0 (0.0%) | 0 (0.0%) | 0 (0.0%) | 0 (0.0%) | 0 (0.0%) | 3 (14.3%) |
|  | Morocco | | 0 (0.0%) | 0 (0.0%) | 0 (0.0%) | 15 (83.3%) | 0 (0.0%) | 1 (4.8%) |
|  | Portugal | | 0 (0.0%) | 0 (0.0%) | 27 (96.4%) | 0 (0.0%) | 0 (0.0%) | 0 (0.0%) |
|  | Switzerland | | 0 (0.0%) | 0 (0.0%) | 0 (0.0%) | 0 (0.0%) | 0 (0.0%) | 1 (4.8%) |
|  | Tunisia | | 0 (0.0%) | 0 (0.0%) | 0 (0.0%) | 0 (0.0%) | 16 (88.9%) | 0 (0.0%) |
|  | Other countries | | 1 (1.7%) | 0 (0.0%) | 1 (3.6%) | 2 (11.1%) | 2 (11.1%) | 4 (19.0%) |
| **Type of practice (n=218)** | University Hospital or Cancer Center | | 10 (17.2%) | 19 (25.3%) | 4 (14.3%) | 10 (55.6%) | 13 (72.2%) | 9 (42.9%) |
|  | General Hospital | | 15 (25.9%) | 12 (16.0%) | 7 (25.0%) | 1 (5.6%) | 0 (0.0%) | 5 (23.8%) |
|  | Private practice | | 17 (29.3%) | 0 (0.0%) | 1 (3.6%) | 6 (33.3%) | 1 (5.6%) | 2 (9.5%) |
|  | Retired | | 1 (1.7%) | 1 (1.3%) | 0 (0.0%) | 0 (0.0%) | 0 (0.0%) | 0 (0.0%) |
|  | Resident | | 15 (25.9%) | 43 (57.3%) | 16 (57.1%) | 1 (5.6%) | 4 (22.2%) | 5 (23.8%) |
|  | Trained pathologist without specific thyroid pathology expertise | | 17 (29.3%) | 21 (28.0%) | 8 (28.6%) | 3 (16.7%) | 8 (44.4%) | 7 (33.3%) |
|  | Trained pathologist with thyroid pathology expertise | | 22 (37.9%) | 11 (14.7%) | 4 (14.3%) | 14 (77.8%) | 6 (33.3%) | 8 (38.1%) |
|  | Thyroid cytopathology expertise only | | 4 (6.9%) | 0 (0.0%) | 0 (0.0%) | 0 (0.0%) | 0 (0.0%) | 1 (4.8%) |

**Supplemental Table 5. Expert consensus on the lesions of interest to evaluate invasion.**

| **All five queries (N = 69)** | | **Two categories (BVI vs. the other four queries) (N = 69)** | |
| --- | --- | --- | --- |
| Certain blood vessel invasion | 26 (37.7%) | Certain blood vessel invasion | 26 (37.7%) |
| Certain capsular invasion | 5 (7.2%) | No certain blood vessel invasion | 43 (62.3%) |
| Doubtful capsular invasion | 2 (2.9%) |  |  |
| Doubtful blood vessel invasion | 13 (18.8%) |  |  |
| No invasion | 23 (33.3%) |  |  |
| **Exclusion of 30 lesions with least expert consensus (N = 39)** | | | |
| **All five queries with exclusion of 30 lesions with least expert consensus (N = 39)** | | **Two categories (BVI vs. the other four queries) with exclusion of 30 lesions with least expert consensus (N = 39)** | |
| Certain blood vessel invasion | 16 (41.0%) | Certain blood vessel invasion | 16 (41.0%) |
| Certain capsular invasion | 4 (10.3%) | No certain blood vessel invasion | 23 (59.0%) |
| Doubtful capsular invasion | 0 |  |  |
| Doubtful blood vessel invasion | 1 (2.6%) |  |  |
| No invasion | 18 (46.1%) |  |  |

**Supplemental Table 6: Interobserver agreement in the assessment of invasion for each individual query among participants with 69/69 of lesions scored at rounds 1, 2 & 3 (211 participants).**

|  | | **Kappa value** | **Z score** | **Probability** |
| --- | --- | --- | --- | --- |
| **Round 1** | Certain CI | 0.2578 | 318.71 | 0.0000 |
|  | Doubtful CI | 0.0858 | 106.13 | 0.0000 |
|  | Certain BVI | 0.3303 | 408.39 | 0.0000 |
|  | Doubtful BVI | 0.1017 | 125.78 | 0.0000 |
|  | No invasion | 0.1890 | 233.69 | 0.0000 |
| **Round 2** | Certain CI | 0.2817 | 348.28 | 0.0000 |
|  | Doubtful CI | 0.1315 | 162.58 | 0.0000 |
|  | Certain BVI | 0.4229 | 522.88 | 0.0000 |
|  | Doubtful BVI | 0.0671 | 82.96 | 0.0000 |
|  | No invasion | 0.2317 | 286.42 | 0.0000 |
| **Round 3** | Certain CI | 0.2905 | 359.12 | 0.0000 |
|  | Doubtful CI | 0.1148 | 141.98 | 0.0000 |
|  | Certain BVI | 0.4001 | 494.70 | 0.0000 |
|  | Doubtful BVI | 0.0700 | 86.50 | 0.0000 |
|  | No invasion | 0.2277 | 281.48 | 0.0000 |

**Supplemental Table 7.  Participant results - Evolution of the kappa and the overall agreement with the expert consensus between rounds, rounds 1, 2 & 3.**

| **211 participants with 69/69 lesions scored at rounds 1, 2 & 3** | | **Differences in kappa coefficient** | | **Difference in overall agreement** | |
| --- | --- | --- | --- | --- | --- |
|  |  | **All five queries** | **Two categories (BVI vs. the other four queries)** | **All five queries** | **Two categories (BVI vs. the other four queries)** |
| Round 1 and 2: | Decreased | 41 (19.4%) | 44 (20.9%) | 27 (12.8%) | 52 (24.6%) |
|  | Stable | 0 (0%) | 1 (0.5%) | 7 (3.3%) | 21 (10.0%) |
|  | Increased | 170 (80.6%) | 166 (78.7%) | 177 (83.9%) | 138 (65.4%) |
| Round 1 and 3: | Decreased | 48 (22.7%) | 43 (20.4%) | 31 (14.7%) | 50 (23.7%) |
|  | Stable | 0 (0%) | 1 (0.5%) | 10 (4.7%) | 17 (8.1%) |
|  | Increased | 163 (77.3%) | 167 (79.1%) | 170 (80.6%) | 144 (68.2%) |
| Round 2 and 3: | Decreased | 119 (56.4%) | 116 (55.0%) | 114 (54.0%) | 100 (47.4%) |
|  | Stable | 1 (0.5%) | 7 (3.3%) | 19 (9.0%) | 34 (16.1%) |
|  | Increased | 91 (43.1%) | 88 (41.7%) | 78 (37.0%) | 77 (36.5%) |

**Supplemental Table 8. Participant results - Agreement with the expert consensus on the thirty lesions that were excluded for low consensus among experts.**

| **252 participants with at least 80% of lesions scored at rounds 1, 2 & 3** | | | **All five queries** | **Two categories (BVI vs. the other four queries)** |  |
| --- | --- | --- | --- | --- | --- |
| Agreement with expert consensus | Round 1 | K median [range] | 0.077 [0.134: 0.350] | 0.194 [-0.154: 0.583] |  |
|  |  | OA (%) median [range] | 26.7 [3.3:  50] | 63.3 [48.1:  80.0] |  |
|  | Round 2 | K median [range] | 0.159 [-0.089: 0.537] | 0.306 [-0.111: 0.722] |  |
|  |  |  |  |  |  |
|  |  | OA (%) median [range] | 36.7 [13.3:  66.7] | 66.7 [46.7:  86.7] |  |
|  | Round 3 | K median [range] | 0.146 [-0.096: 0.534] | 0.286 [-0.129: 0.783] |  |
|  |  |  |  |  |  |
|  |  | OA (%) median [range] | 33.3 [10.0:  66.7] | 66.7 [46.7:  90.0] |  |

**Supplemental Table 9. Participant results after exclusion of the 30 lesions with least expert consensus - Intraobserver and interobserver agreement among the participants and agreement of the participants with the expert consensus.**

| **252 participants with at least 80% of lesions scored at rounds 1, 2 & 3; N lesions = 39** | | | **All five queries** | **Two categories (BVI vs. the other four queries)** |
| --- | --- | --- | --- | --- |
| Intraobserver agreement | | K median [range] | 0.408 [0.114: 0.691] | 0.588 [0.131: 0.887] |
|  |  | OA (%) median [range] | 41.0 [10.3:  71.8] | 74.4 [41.0: 92.3] |
| Interobserver agreement | Round 1 | Combined K value | 0.247 | 0.410 |
|  |  | OA [95% CI] (%) | - | - |
|  | Round 2 | Combined K value | 0.315 | 0.539 |
|  |  | OA [95% CI] (%) | - | - |
|  | Round 3 | Combined K value | 0.31 | 0.524 |
|  |  | OA [95% CI] (%) | - | - |
| Agreement with expert consensus | Round 1 | K median [range] | 0.358 [0.033 0.705] | 0.506 [0.027: 0.945] |
|  |  | OA (%) median [range] | 53.8 [12.8: 79.5] | 79.5 [59.0: 97.4] |
|  | Round 2 | K median [range] | 0.431 [0.102: 0.802] | 0.655 [0.027: 0.945] |
|  |  | OA (%) median [range] | 59 [23.1: 87.2] | 84.6 [61.5: 97.4] |
|  | Round 3 | K median [range] | 0.439 [0.065: 0.851] | 0.638 [0.061: 0.945] |
|  |  | OA (%) median [range] | 59 [23.1: 89.7] | 82.5 [61.5: 97.4] |

**Supplemental Table 10.  Expert results** **after exclusion of the 30 lesions with least expert consensus - Intraobserver and interobserver agreement among experts and agreement of each expert with the expert consensus.**

| **N lesions = 39** | | | | **All five queries** | **Two categories (BVI vs. the other 4 queries)** |
| --- | --- | --- | --- | --- | --- |
| Intraexpert agreement | | | K | Between 0.635 (95% IC [0.445 : 0.807]) and 0.957 (95% IC [0.860 : 1]) | Between 0.762 (95% IC [0.533 : 0.945]) and 1 |
|  |  |  | OA | Between 74.4% (95% IC [57.9 ; 87.0)  and 97.4% (95% IC[86.5 ; 99.9]) | Between 89.7% (95% IC [75.8 : 97.1]) and 100% (95% IC [91.0 : 100.0) |
| Interexpert agreement | Round 1 | | K [95% CI] | 0.587 [0.465: 0.718] | 0.812 [0.649 : 0.957] |
|  |  |  | OA [95% CI] | 59% [42.1 : 74.4] | 87.2% [72.6 : 95.7] |
|  | Round 2 | | K [95% CI] | 0.684 [541 : 0.826] | 0.808 [0.644 : 0.951] |
|  |  |  | OA [95% CI] | 69.2% [52.4 : 83.0] | 87.2% [72.6 : 95.7] |
| Agreement with expert consensus | Round 1 | Expert 1 | K [95% CI] | 0.701 [0.526 : 0.871] | 0.889 [0.669 : 1.000] |
|  |  |  | OA (%) [95% CI] | 79.5 [63.5 : 90.7] | 94.9 [82.7 : 99.4] |
|  |  | Expert 2 | K [95% CI] | 0.839 [0.670 : 0.958] | 1 [ : ] |
|  |  |  | OA (%) [95% CI] | 89.7 [75.8 : 97.1] | 100.0 [91.0 : 100.0] |
|  |  | Expert 3 | K [95% CI] | 0.706 [0.526 : 0.886] | 0.83 [0.616 : 1.000] |
|  |  |  | OA (%) [95% CI] | 79.5 [63.5 : 90.7] | 92.3 [79.1 : 98.4] |
|  | Round 2 | Expert 1 | K [95% CI] | 0.849 [0.712 : 0.962] | 0.762 [0.559 : 0.943] |
|  |  |  | OA (%) [95% CI] | 89.7 [75.8 : 97.1] | 89.7 [75.8 : 97.1] |
|  |  | Expert 2 | K [95% CI] | 0.879 [0.723 : 1.000] | 1 [ : ] |
|  |  |  | OA (%) [95% CI] | 92.3 [79.1 : 98.4] | 100.0 [91.0 : 100.0] |
|  |  | Expert 3 | K [95% CI] | 0.779 [0.613 : 0.926] | 0.945 [0.796 : 1.000] |
|  |  |  | OA (%) [95% CI] | 84.6 [69.5 : 94.1] | 97.4 [86.5 : 99.9] |

**Supplemental Table 11.** **Comparison of the agreement with the expert consensus between trained pathologists and pathology residents and among Italian, Portuguese, and French speaking countries sessions, rounds 1, 2 & 3.**

| **252 participants with at least 80% of lesions scored at rounds 1, 2 & 3** | | | **Trained pathologists** | **Residents** | ***p- value*** | **French speaking countries session** | **Italian session** | **Portuguese session** | ***p-value*** |
| --- | --- | --- | --- | --- | --- | --- | --- | --- | --- |
| N | | | 134 | 84 |  | 128 | 89 | 35 |  |
| **All five queries** | K median  [range] | Round1 | 0.253  [0.050: 0.494] | 0.209  [0.034: 0.469] | 0.0027 | 0.251  [0.034: 0.494] | 0.212  [0.071: 0.439] | 0.241  [0.092: 0.487] | > 0.05 |
|  |  | Round2 | 0.354  [0.067: 0.588] | 0.275  [0.070: 0.572] | <0.0001 | 0.339  [0.067: 0.581] | 0.299  [0.058: 0.588] | 0.342  [0.091: 0.472] |  |
|  |  | Round3 | 0.343  [0.099: 0.702] | 0.271  [0.071: 0.586] | <0.0001 | 0.330  [0.071: 0.586] | 0.300  [0.088: 0.603] | 0.317  [0.075: 0.702] |  |
|  | OA (%) median [range] | Round1 | 42  [17.4: 62.3] | 38.4  [13.0: 60.9] | <0.0001 | 42  [13.0: 62.3] | 39.1  [21.7: 58.0] | 40.6  [27.5: 62.3] | 0.2499 |
|  |  | Round2 | 52.2  [29.0: 71.0] | 44.9  [24.6: 69.6] | <0.0001 | 50.7  [30.4: 69.6] | 46.4  [21.7: 71.0] | 50.7  [29.0: 62.3] | 0.0224 |
|  |  | Round3 | 51.4  [23.2: 78.3] | 44.9  [21.7: 69.6] | <0.0001 | 50.4  [27.5: 69.6] | 46.4  [27.5: 72.5] | 49.3  [21.7: 78.3] | 0.1501 |
| **Two categories (BVI vs. the other 4 queries)** | K median  [range] | Round1 | 0.368  [0.026: 0.753] | 0.357  [0.094: 0.782] | > 0.05 | 0.358  [0.026: 0.720] | 0.368  [0.094: 0.691] | 0.356  [0.111: 0.782] | > 0.05 |
|  |  | Round2 | 0.516  [0.111: 0.728] | 0.464  [0.114: 0.741] | 0.0116 | 0.501  [0.170: 0.728] | 0.495  [0.155: 0.741] | 0.471  [0.111: 0.682 |  |
|  |  | Round3 | 0.500  [0.009: 0.782] | 0.432  [0.117: 0.745] | 0.0039 | 0.500  [0.009: 0.782] | 0.466  [0.185: 0.728] | 0.483  [0.117: 0.701] |  |
|  | OA (%) median [range] | Round1 | 72.8  [60.9: 88.4] | 72.5  [62.3: 89.9] | > 0.05 | 72.5  [60.9: 87.0] | 72.5  [62.3: 85.5] | 73.9  [63.8: 89.9] | > 0.05 |
|  |  | Round2 | 78.1  [65.2: 87.0] | 76.1  [62.3: 88.4] | 0.0298 | 76.8  [62.3: 87.0] | 76.8  [63.8: 88.4] | 76.8  [63.8: 85.5] |  |
|  |  | Round3 | 76.8  [60.9: 89.9] | 75.4  [60.9: 88.4] | 0.0220 | 76.8  [60.9: 89.9] | 75.4  [62.3: 87.0] | 76.8  [60.9: 85.5] |  |

**Supplemental Table 12.** **Comparison of the agreement with the expert consensus according to the type of practice among trained pathologists (excluding residents), rounds 1, 2 & 3.**

|  | | | **University Hospital or Cancer center** | **General Hospital** | **Private practice** | ***p-value*** |  |
| --- | --- | --- | --- | --- | --- | --- | --- |
|  |  |  |  |  |  |  |  |
| N | | | 65 | 40 | 27 |  |  |
| **All five queries** | K median [range] | Round 1 | 0.257 [0.050: 0.494] | 0.253 [0.053: 0.454] | 0.256 [0.096: 0.396] | > 0.05 |  |
|  |  | Round 2 | 0.371 [0.067: 0.588] | 0.348 [0.120: 0.520] | 0.368 [0.173: 0.487] |  |  |
|  |  | Round 3 | 0.344 [0.099: 0.603] | 0.339 [0.129: 0.702] | 0.352 [0.101: 0.526] |  |  |
|  | OA (%)  median [range] | Round 1 | 43.5 [17.4: 62.3] | 42.8 [23.2: 59.4] | 40.6 [24.6: 53.6] | > 0.05 |  |
|  |  | Round 2 | 53.6 [29.0: 71.0] | 52.2 [33.3: 62.2] | 53.6 [29.0: 71.0] |  |  |
|  |  | Round 3 | 52.2 [23.2: 72.5] | 50.7 [33.3: 65.2] | 52.2 [29.0: 66.7] |  |  |
| **Two categories (BVI vs. the other 4 queries)** | K median [range] | Round 1 | 0.401 [0.026: 0.753] | 0.344 [0.094: 0.653] | 0.368 [0.143: 0.569] | > 0.05 |  |
|  |  | Round 2 | 0.547 [0.111: 0.728] | 0.499 [0.170: 0.716] | 0.519 [0.274: 0.720] |  |  |
|  |  | Round 3 | 0.506 [0.185: 0.782] | 0.470 [0.253: 0.691] | 0.541 [0.009: 0.779] |  |  |
|  | OA (%) median [range] | Round 1 | 73.9 [60.9: 88.4] | 72.5 [63.8: 84.1] | 72.5 [63.1: 81.2] | > 0.05 |  |
|  |  | Round 2 | 78.3 [65.2: 87.0] | 77.4 [66.7: 87.0] | 76.8 [66.7: 71.0] |  |  |
|  |  | Round 3 | 76.8 [65.2: 89.9] | 50.7 [33.3: 65.2] | 79.7 [60.9: 89.9] |  |  |

**Supplemental Table 13. Comparison of the agreement with the expert consensus according to the number of thyroid surgical pathology cases diagnosed per year among trained pathologists (excluding residents), rounds 1, 2 & 3.**

|  | | | **< 40 thyroid surgical pathology cases signed out per year^a^** | **> 40 thyroid surgical pathology cases signed out per year^a^** | ***p- value*** |  |
| --- | --- | --- | --- | --- | --- | --- |
|  |  |  |  |  |  |  |
| N | | | 67 | 67 |  |  |
| **All five queries** | K median  [range] | Round 1 | 0.252 [0.053: 0.454] | 0.258 [0.050: 0.494] | > 0.05 |  |
|  |  | Round 2 | 0.348 [0.120: 0.493] | 0.368 [0.067: 0.588] |  |  |
|  |  | Round 3 | 0.329 [0.129: 0.702] | 0.350 [0.099: 0.603] |  |  |
|  | OA (%) median  [range] | Round 1 | 42 [23.2: 59.4] | 43.5 [17.4: 62.3] | > 0.05 |  |
|  |  | Round 2 | 51.5 [29.0: 65.2] | 53.6 [31.9: 71.0] |  |  |
|  |  | Round 3 | 50.7 [33.3: 78.3] | 52.2 [23.2: 72.5] |  |  |
| **Two categories (BVI vs. the other 4queries)** | K median  [range] | Round 1 | 0.368 [0.094: 0.720] | 0.360 [0.026: 0.753] | > 0.05 |  |
|  |  | Round 2 | 0.511 [0.111: 0.779] | 0.519 [0.170: 0.728] |  |  |
|  |  | Round 3 | 0.502 [0.185: 0.779] | 0.499 [0.009: 0.782] |  |  |
|  | OA (%) median [range] | Round 1 | 72.5 [63.8: 87.0] | 73.9 [60.9: 88.4] | > 0.05 |  |
|  |  | Round 2 | 78.3 [65.2: 87.0] | 78.0 [66.7: 87.0] |  |  |
|  |  | Round 3 | 76.8 [65.2: 89.9] | 76.8 [60.9: 89.9] |  |  |

^a^The 40 thyroid surgical pathology case cut-off corresponds to the median number of cases signed out by trained pathologists.

**Supplemental Table 14. Comparison of the agreement with the expert consensus according to the number of years of practice among trained pathologists (excluding residents), rounds 1, 2 & 3.**

|  | | | **< 17.5 years of practice^a^** | **> 17.5 years of practice^a^** | ***p- value*** |  |
| --- | --- | --- | --- | --- | --- | --- |
|  |  |  |  |  |  |  |
| N | | | 67 | 67 |  |  |
| **All five queries** | K median [range] | Round 1 | 0.251 [0.050: 0.487] | 0.256 [0.071: 0.494] | > 0.05 |  |
|  |  | Round 2 | 0.350 [0.120: 0.588] | 0.368 [0.067: 0.520] |  |  |
|  |  | Round 3 | 0.344 [0.099: 0.702] | 0.342 [0.101: 0.603] |  |  |
|  | OA (%) median [range] | Round 1 | 42 [17.4: 62.3] | 42.0 [27.5: 62.3] | > 0.05 |  |
|  |  | Round 2 | 52.2 [29.0: 71.0] | 53.6 [31.9: 65.2] |  |  |
|  |  | Round 3 | 52.2 [23.2: 78.3] | 50.7 [29.0: 72.5] |  |  |
| **Two categories (BVI vs. the other 4 queries)** | K median [range] | Round 1 | 0.368 [0.026: 0.753] | 0.368 [0.094: 0.696] | > 0.05 |  |
|  |  | Round 2 | 0.503 [0.111: 0.728] | 0.519 [0.170: 0.720] |  |  |
|  |  | Round 3 | 0.502 [0.185: 0.779] | 0.499 [0.009: 0.782] |  |  |
|  | OA (%) median [range] | Round 1 | 73.1 [60.9: 88.4] | 72.5 [63.8: 85.5] | > 0.05 |  |
|  |  | Round 2 | 76.8[65.2: 89.9] | 78.3 [66.7: 87.0] |  |  |
|  |  | Round 3 | 76.8[65.2: 89.9] | 76.8 [60.9: 89.9] |  |  |

^a^The 17.5 years cut-off corresponds to the median of the years of practice for trained pathologists.
